# Shear bond strength of three composite resins for orthodontic bracket bonding on enamel and ceramic surfaces: an in vitro study after accelerated aging

**DOI:** 10.2340/biid.v13.46209

**Published:** 2026-06-25

**Authors:** Melina Nohemi Atarihuana Diaz, Mauricio Aguirre Balseca, Marjory Elizabeth Vaca Zapata, Karina Maria Salvatore Freitas, Krisnaya Muñoz

**Affiliations:** a Department of Orthodontics, University of the Hemispheres, Quito, Ecuador; b Deparment of Endodontics, University of the Hemispheres, Quito, Ecuador; c Ingá University Center UNINGA. Maringá, Brazil

**Keywords:** Shear bond strength, orthodontic brackets, composite resins

## Abstract

**Objective:**

To evaluate and compare the shear bond strength (SBS) of three composite resins, Z250 (3M), Transbond XT (3M), and Brace Paste (AO), used for orthodontic bracket bonding on human enamel and ceramic surfaces, after undergoing accelerated aging.

**Materials and Methods:**

An in vitro experimental design was conducted using 30 enamel specimens (human premolars) and 30 ceramic specimens (Brava Block, FGM), divided into three subgroups per substrate (*n* = 10 each). All specimens were etched (37% phosphoric acid for enamel, 9.6% hydrofluoric acid for ceramic), treated with silane (ceramics), and bonded using the designated composite resins. Specimens were subjected to an autoclave aging protocol. SBS was measured using a universal testing machine, and statistical analysis included Analysis of Variance (ANOVA), *t* tests, and chi-square tests (α = 0.05).

**Results:**

Transbond XT exhibited the highest mean SBS on ceramic surfaces (10.4 ± 5.2 MPa), while Brace Paste showed the highest SBS on enamel (7.3 ± 2.4 MPa). However, no statistically significant differences were observed among the composite resins in either substrate (*p* > 0.05). All materials demonstrated SBS values within or close to the clinically acceptable range for orthodontic bonding (approximately 6–8 MPa).

**Conclusion:**

Although numerical differences were found, all three composite resins demonstrated comparable performance. When appropriate surface conditioning protocols are applied, any of the tested materials can be considered reliable for bracket bonding to both enamel and ceramic substrates. Further in vivo studies are recommended to validate long-term durability.

## Introduction

The evolution of dental materials has followed the increasing clinical and aesthetic demands of modern dentistry, driving the development of more efficient, versatile, and cost-effective adhesive systems [1, 2]. From early epoxy adhesives to contemporary Bis-GMA-based (GMA: glycidyl methacrylate )composites, direct and indirect bonding in orthodontics has undergone substantial technological advancements [3, 4]. In orthodontic practice, the choice of bonding material is critical to ensure proper bracket retention to the enamel, especially under tensile and shear forces throughout treatment [5–7].

A wide range of resin-based materials is currently available, including conventional composites, resin-modified glass ionomers, and dual-cure adhesives, each offering specific mechanical and handling properties [8, 9]. Their organic matrix typically contains monomers such as Bis-GMA, urethane dimethacrylate (UDMA), and triethylene glycol dimethacrylate (TEGDMA), combined with initiator systems like camphorquinones or tertiary amines, which influence viscosity, clinical manipulation, and long-term color stability [10–12]. The inorganic filler phase, composed of silica, quartz, zirconia, and barium, plays a central role in mechanical reinforcement, translucency, and reduction of polymerization shrinkage [10, 13].

Among the most widely used materials in clinical orthodontics are light-cured resins and dual-cure resin cements, which demonstrate excellent adhesion even in humid environments and in areas with limited light exposure [3, 14]. Nevertheless, selecting the ideal adhesive system remains a clinical challenge, as it must be tailored to individual patient needs, technique sensitivity, and biomechanical demands [6, 15]. Moreover, hydrothermal aging and intraoral wear can compromise long-term bond strength, underscoring the need for comparative studies on material durability [16–18].

Orthodontic brackets are most commonly bonded to enamel surfaces. However, the increasing prevalence of ceramic and resin-based computer-aided design/computer-aided manu-facturing (CAD/CAM) restorations in adult patients has made bonding to indirect restorative substrates an increasingly relevant clinical challenge. Bonding to these materials remains particularly difficult due to differences in surface composition, microstructure, and surface energy compared with natural enamel. While enamel provides a predictable substrate after phosphoric acid conditioning, ceramic materials and resin-based CAD/CAM blocks often require more complex surface treatments, such as hydrofluoric acid etching followed by silanization, to achieve adequate micromechanical and chemical retention. These interfaces are also more susceptible to hydrothermal degradation over time, leading to higher rates of bracket failure in patients with extensive prosthetic rehabilitation.

Recent comparative studies have reported variable shear bond strength (SBS) on ceramic substrates, with some adhesive systems performing adequately on enamel but exhibiting reduced stability on ceramic surfaces after thermocycling or artificial aging [5, 14, 19, 20]. Understanding these differences is essential, as orthodontists increasingly face mixed-substrate bonding scenarios. Therefore, evaluating the performance of commonly used composite resins on both enamel and ceramic surfaces under accelerated aging conditions provides clinically relevant information for substrate-specific material selection.

Furthermore, recent in vitro studies have demonstrated that zirconia-based ceramics present significantly lower bond strength for both metal and ceramic brackets compared to enamel, especially after artificial aging or thermocycling, highlighting the risk of bond failure in mixed-substrate orthodontic cases [21–23].

Indirect, laboratory-based bonding techniques have gained popularity because they allow more accurate bracket positioning under controlled conditions, reduce chair time, and minimize saliva contamination during clinical bonding procedures, potentially decreasing adhesive failures [24]. However, the effectiveness of the adhesive system remains dependent on its resistance to aging and functional stress [16, 19].

Given the diversity of available adhesive systems and the need for comparative evidence, this in vitro study aimed to evaluate and compare the SBS of three commonly used composite resins, Z250, Transbond XT™, and Brace Paste™, for bracket bonding to enamel and resin-based CAD/CAM material after artificial aging. The goal was to provide clinically relevant data that may assist in material selection based on mechanical performance.

## Material and methods

This study was approved by the Ethics Committee of the University of the Hemispheres, Quito, Ecuador (protocol n. CEUHE25-92). This study aims to evaluate the adhesive strength of different composite resins used in the bonding of orthodontic brackets to two types of surfaces: ceramic (resin-based CAD/CAM blocks) and enamel (human teeth). Through an in vitro experimental methodology, both the mechanical properties of the tested resins and their performance under controlled aging conditions were assessed.

## Experimental design

### Study population and specimens

Group Ceramic: Thirty ceramic specimens made from Brava Block (FGM, Joinville, Brazil) resin-based CAD/CAM blocks were used, cut, and prepared, measuring 14 mm × 14 mm × 3 mm, and divided into three subgroups of ten specimens each.

Group Enamel: Thirty extracted human premolars were selected (ten per group). All teeth were caries-free, unrestored, and had not undergone endodontic treatment.

### Specimens preparation

All procedures were performed by a single calibrated operator to minimize procedural variability.

### Ceramic

**Figure 1:**
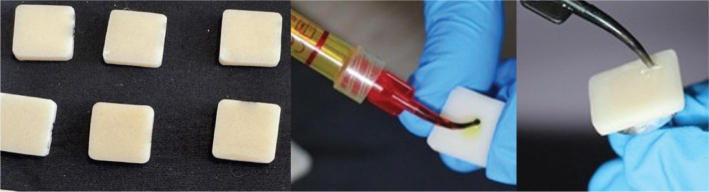
(A) Ceramic; (B) Surface conditioning of the ceramic with 9.6% hydrofluoric acid; (c) Application of silane on the surfaces of the ceramic.

Plates were sectioned from Brava Blocks at 250 rpm using a diamond disk and continuous irrigation. Final dimensions: 14 mm × 14 mm × 3 mm (Figure 1A).

Surfaces were sequentially polished using 600-, 1,000-, and 1,200-grit silicon carbide papers and stored in sterile plastic containers until use. Surface conditioning of the resin-based CAD/CAM material (Brava Block, FGM, Joinville, Brazil) was performed according to the manufacturer’s recommendations and previous studies [25, 26]. The ceramic surfaces were etched with 9.6% hydrofluoric acid (ULTRADENT, South Jordan, USA) for 20 s (Figure 1B), rinsed with water for 20 s, and silanized using a microbrush with silane coupling agent (ULTRADENT, South Jordan, USA) (Figure 1C), followed by air drying for 60 s at 30 PSI.

### Enamel

**Figure 2:**
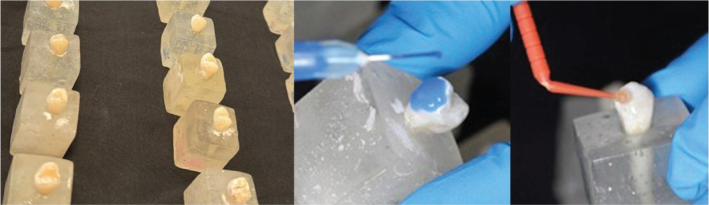
(A) Enamel (human premolars); (B) Etching of the enamel with 37% phosphoric acid; (C) Adhesive application with a microbrush.

Healthy premolars extracted for orthodontic or periodontal reasons were collected from private clinics and stored in 0.9% saline solution at room temperature (20–25°C) until use (Figure 2A). Prior to bonding, the enamel surfaces were rinsed, dried with sterile gauze, and cleaned with pumice and 5.25% sodium hypochlorite using a prophylactic brush for 1 min. Enamel conditioning was then performed with 37% phosphoric acid (ULTRADENT) for 15 s (Figure 2B), followed by water rinsing and air drying at 30 PSI.

### Bracket bonding

Conventional metal brackets (Orthometric, Roth prescription, 0.022” × 0.018”, Marília, Brazil) were used. These brackets are manufactured using metal injection molding (MIM) technology with 17-4 PH stainless steel. The bracket base features an 80-micron mesh laser-welded to the anatomical base, with retention pins to enhance adhesive performance.

For enamel, premolar brackets were used; for ceramic, incisor brackets were used. A positioning template was created to standardize bracket placement on ceramic specimens. For enamel specimens, the center of the clinical crown was determined visually based on the mesiodistal width and occlusogingival height of the buccal surface to standardize bracket positioning.

The following composite resins and corresponding primers/adhesive systems were tested (Table 1):

**Table 1:** Materials used in the study and their respective manufacturers and clinical applications.

Material	Manufacturer	Type/Application
Brava Block	FGM	Resin-based CAD/CAM block
Z250 composite	3M ESPE	Composite restorative material
Adper Single Bond 2 adhesive	3M ESPE	Fifth-generation etch-and-rinse adhesive
Transbond XT composite	3M Unitek	Orthodontic light-cured composite
Transbond XT Primer	3M Unitek	Orthodontic primer
Brace Paste composite	American Orthodontics	Orthodontic composite
MTP Primer	American Orthodontics	Orthodontic primer
Hydrofluoric acid 9.6%	Ultradent	Ceramic surface conditioning
Phosphoric acid 37%	Ultradent	Enamel etching
Silane coupling agent	Ultradent	Ceramic silanization
Metal brackets	Orthometric	Stainless steel orthodontic brackets
LED curing unit (WOODPECKER II)	Woodpecker	Light polymerization
Universal testing machine (MTS T5002)	MTS Systems Corporation	Shear bond strength testing

- Group Z250—Adper Single Bond 2 adhesive + Z250 composite (3M ESPE, St. Paul, USA)
- Group Transbond XT—Transbond XT primer + Transbond XT composite (3M Unitek, Monrovia, USA)
- Group Brace Paste—MTP Primer + Brace Paste composite (American Orthodontics, Sheboygan, USA)

All adhesives were applied with a microbrush (Figure 2C) and light-cured under identical conditions using the same light-emitting diode (LED) curing unit.

The output intensity of the LED curing unit (WOODPECKER II, Guilin Woodpecker Medical Instrument Co., Guilin, Guangxi, China) was verified prior to specimen preparation using a calibrated dental radiometer (LM-1 Curing Light Meter, Guilin Woodpecker Medical Instrument Co., Guilin, Guangxi, China) to ensure consistency within the manufacturer-reported range (2,700–3,000 mW/cm²). The light tip was positioned perpendicular to the bracket surface at an approximate distance of 2 mm and maintained in a standardized orientation during polymerization. Light curing was performed through the bracket wings from four directions (mesial, distal, occlusal, and gingival), 3 s per direction, totaling 12 s of exposure. This protocol was standardized for all specimens to minimize variability in light transmission and ensure uniform polymerization.

**Figure 3:**
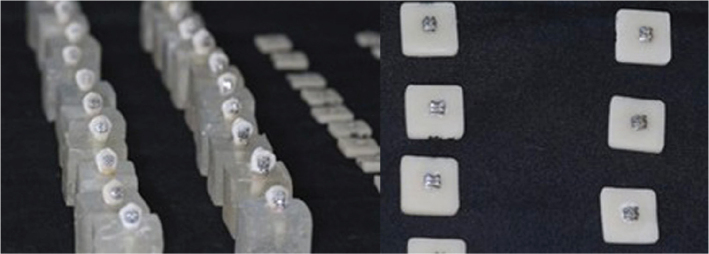
Brackets bonded to enamel and to ceramic.

Brackets were positioned using a bracket holder (MORELLI, Sorocaba, Brazil), pressed to eliminate bubbles, and excess resin was removed (Figure 3).

### Aging protocol

All specimens were initially stored in distilled water at room temperature for 24 h prior to aging. Accelerated hydrothermal aging was subsequently performed using an autoclave protocol at temperatures ranging from 123 to 133°C and pressure between 18.3 and 29.8 psi. Five cycles consisting of 4 h of steam exposure followed by 4 h of rest were applied, totaling 40 h of aging [27–29].

### SBS test and adhesion evaluation

After aging, specimens underwent SBS testing in a universal testing machine operated by a single examiner.

SBS was calculated using the formula:

SBS (MPa) = F/A

where F represents the maximum force at failure (in Newtons) and A corresponds to the bracket base area (in mm²).

Since two different bracket types were used, the actual base areas provided by the manufacturer were considered for each calculation. The premolar brackets presented a base area of 13.30 mm², while the incisor brackets used for the ceramic specimens had a base area of 9.75 mm². These values were used directly in the calculation of SBS.

### SBS test

**Figure 4:**
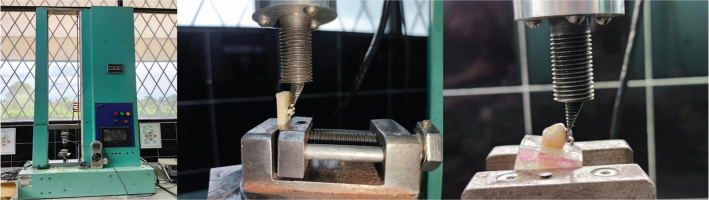
(A) MTS universal testing machine, model T5002; (B) Stabilization and fixation of the acrylic base in the testing machine and positioning of the stainless-steel beveled tip; (C) Direct fixation of the ceramic specimen in the testing machine and positioning of the stainless steel beveled tip.

After completing the cementation and conditioning phases for both Group Ceramic and Group Enamel, the shear testing phase was carried out. The machine used in this study was the MTS T5002 universal testing machine, operated by a single operator (Figure 4A).

For the premolar group, each specimen was embedded in a transparent acrylic base, which was positioned in a mini clamp to ensure accurate placement at an appropriate angle, avoiding interference from the dental structure (Figure 4B).

In contrast, Group Ceramic was fixed directly onto the central axis of the mini clamp at a 90-degree angle, due to the absence of natural dental curvature. This setup ensured precise and stable positioning. Once the specimens were secured, a controlled pressure force was applied using a stainless steel beveled tip, 1 mm thick and 10 mm wide (Figure 4C). The machine was calibrated in Newtons, and the force values were directly recorded by the pressure sensor.

### Statistical analysis

The data obtained from the bond strength measurements (in MPa) were organized into tables and analyzed using SPSS software.

Prior to inferential analysis, data normality was assessed using the Shapiro–Wilk test. As all groups showed normal distribution (*p* > 0.05), parametric tests were applied. One-way ANOVA was used to compare SBS among the three adhesive systems within each substrate, followed by Tukey post hoc tests. Student’s *t* tests were used to compare substrates (human enamel vs. ceramic). Effect sizes were calculated to assess the magnitude of differences, using η² (eta squared) for ANOVA and Cohen’s d for *t* tests. The level of significance was set at α = 0.05.

## Results

SBS testing on enamel varied from 7.3 ± 2.4 MPa (Brace Paste) to 9.4 ± 3.5 MPa (Transbond XT), and on ceramic from 6.5 ± 2.4 (Brace Paste) to 10.4 ± 5.2 MPa (Transbond XT). Transbond XT demonstrated the highest mean SBS values numerically, and Brace Paste, the lowest; however, no statistically significant differences were observed among the materials (*p* > 0.05; Table 2). Effect size analysis revealed a small-to-moderate effect for human enamel (η² ≈ 0.1) and a moderate effect for ceramic substrates (η² ≈ 0.2), indicating that substrate-related variability may have influenced the magnitude of differences despite the absence of statistical significance.

**Table 2:** Descriptive statistics (mean ± SD, MPa) and one-way ANOVA for shear bond strength of the three composite resins on human enamel and ceramic substrates (*n* = 10 per subgroup).

Group	Resin	*N*	Mean	SD	95% CI Min	95% CI Max	η²	*p*
Enamel	Transbond XT	10	9.4	3.5	6.9	11.9	0.1	0.237
	Brace Paste	10	7.3	2.4	5.7	9.0		
	Z 250	10	7.5	2.9	5.4	9.6		
Ceramic	Transbond XT	10	10.4	5.2	6.6	14.1	0.2	0.060
	Brace Paste	10	6.5	2.4	4.9	8.2		
	Z 250	10	10.1	3.3	7.7	12.4		

SD: standard deviation; CI: confidence interval.

In Table 3, no significant variation was found among the composite resins. Comparison between substrates showed that Transbond XT and Z250 presented higher SBS values on ceramic, whereas Brace Paste showed higher SBS values on enamel.

**Table 3:** Tukey test.

Dependent variable	Resin	Resin	Mean difference	CI 95% Min	CI 95% Max	*p*
Enamel	Transbond XT	Brace Paste	2.1	−1.2	5.4	0.27
		Z 250	1.9	−1.4	5.2	0.34
	Brace Paste	Z 250	−0.2	−3.5	3.1	0.99
Ceramic	Transbond XT	Brace Paste	3.8	−0.4	8.0	0.08
		Z 250	0.3	−3.9	4.5	0.99
	Brace Paste	Z 250	−3.5	−7.8	0.7	0.11

**Figure 5:**
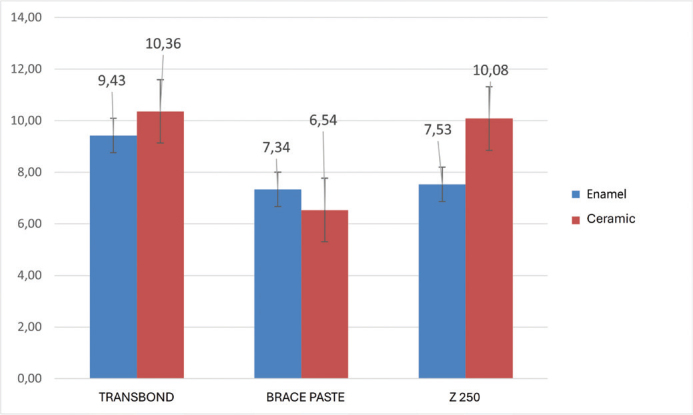
Mean SBS of composite resins in bracket bonding.

Figure 5 illustrates the distribution of SBS values among the three composite resins for bracket bonding. Despite numerical variation in mean values, statistical analysis confirmed that no significant differences were detected among the groups (*p* > 0.05).

Additionally, bond strength was compared between enamel and ceramic using the Student’s *t* test.

In Table 4, no statistically significant differences were found between the composite resins when comparing bond strength on enamel and ceramic. Cohen’s d effect sizes were small for Transbond XT (*d* = 0.2) and Brace Paste (*d* = 0.3), whereas Z250 demonstrated a large effect size (*d* = 0.8), suggesting a substantial magnitude of difference between substrates despite not reaching statistical significance.

**Table 4:** Student *t* test.

Resin	Group	*N*	Mean	SD	Cohen d	*P*
TRANSBOND XT	Enamel	10	9.4	3.5	0.2	0.645
	Ceramic	10	10.4	5.2		
BRACE PASTE	Enamel	10	7.3	2.4	0.3	0.455
	Ceramic	10	6.5	2.4		
Z 250	Enamel	10	7.5	2.9	0.8	0.084
	Ceramic	10	10.1	3.3		

SD: standard deviation.

## Discussion

The present in vitro study evaluated the SBS of three composite resins, Z250 (3M), Transbond XT (3M), and Brace Paste (AO), used for orthodontic bracket bonding on two substrates: enamel (human teeth) and ceramic (Brava Block). While numerical differences in SBS were observed among the resins, no statistically significant differences were found, which suggests clinical flexibility in choosing the adhesive system depending on substrate and operator preference.

Previous studies support the clinical reliability of Transbond XT, which has consistently shown high SBS values in both enamel and indirect bonding situations. For example, a study comparing Transbond XT and Ortholink found higher SBS values for Transbond XT, supporting its widespread clinical use [5].

Another in vitro study comparing Z250, Transbond XT, and other resins also found no significant statistical differences among them, though Transbond XT consistently performed above the 7 MPa clinical threshold suggested for reliable bonding [30].

The numerical performance of Transbond XT in our study aligns with its established reputation as a benchmark resin, consistently demonstrating SBS values above 7 MPa in both enamel and ceramic substrates in prior literature [5, 30]. This trend was also observed in the present results; however, the lack of statistical significance is likely attributable to the relatively high variability, particularly in ceramic groups, and the limited statistical power inherent to a sample size of *n* = 10 per group. Thus, while Transbond XT numerically outperformed the other materials, these differences should be interpreted cautiously.

Regarding Brace Paste, although fewer studies directly evaluate its bond strength, the present study provides useful data for comparison. In our results, Brace Paste showed a higher SBS on enamel compared to the other two composite resins, suggesting it may be a viable choice for direct bonding in clinical settings when combined with proper surface treatment protocols [30].

Although the differences were not statistically significant, the numerical trends may be explained by compositional and rheological characteristics of the resins. Transbond XT contains a relatively high filler content and exhibits greater viscosity, which may enhance its mechanical interlocking with ceramic substrates when combined with hydrofluoric acid etching and silane application, as previously described in adhesive performance studies [5, 14]. In contrast, Brace Paste demonstrated higher values on enamel, possibly due to its lower viscosity and optimized primer formulation, which may facilitate better penetration into enamel microporosities created by phosphoric acid etching. Composite resins such as Z250, which contain a higher inorganic filler load and have a stiffer consistency, tend to show reduced flow into surface irregularities, potentially explaining its intermediate behavior on both substrates [9, 13]. These material-dependent effects, although not statistically significant in this sample, provide clinically relevant insight into how adhesive chemistry and filler morphology may influence retention on different substrates.

As expected, differences in bond strength were observed between substrates. Bonding to etched human enamel with phosphoric acid and a fifth-generation adhesive showed distinct performance when compared to ceramic surfaces etched with hydrofluoric acid and silanized, which is consistent with the literature [20].

It is important to emphasize that the “ceramic” substrate evaluated in this study corresponds to a resin-based CAD/CAM material (Brava Block), which differs substantially from conventional glass ceramics and polycrystalline zirconia in composition, microstructure, and bonding behavior. Unlike glass ceramics, in which hydrofluoric acid selectively dissolves the glassy matrix to create micromechanical retention, resin-based CAD/CAM materials contain a polymeric matrix with dispersed inorganic fillers, and hydrofluoric acid conditioning primarily affects the filler–matrix interface. Consequently, the present findings should not be directly extrapolated to conventional ceramic restorations, and their interpretation should be restricted to resin-based CAD/CAM materials subjected to similar surface treatment protocols.

Additionally, the accelerated aging protocol via autoclave simulated oral environmental stress, aiming to assess the durability of these composite resins under hydrothermal degradation. The use of an accelerated aging protocol in an autoclave simulating hydrothermal stress revealed that bond strength values remained within clinically acceptable limits, even after exposure to high temperatures and pressure. This corroborates the findings of Meguro et al. [31], who showed that thermocycling and moisture exposure can degrade adhesive interfaces but not necessarily compromise clinical effectiveness if the adhesive system is robust. Previous study also confirms that temperature cycling and humidity can degrade the adhesive interface, weakening long-term mechanical performance [5].

Despite this degradation, all three composite resins demonstrated SBS values broadly compatible with those traditionally referenced in the literature, although contemporary reviews caution that such thresholds should not be interpreted rigidly, given the multifactorial nature of clinical bond failures [32, 33].

Although accelerated aging in an autoclave provides a controlled hydrothermal challenge, it does not replicate physiologic intraoral conditions. Temperatures of 123–133°C combined with high-pressure saturated steam induce degradation pathways that are more aggressive than those typically encountered clinically or in conventional artificial aging protocols such as thermocycling (5–55°C) or long-term water storage [27–29]. The purpose of employing this protocol was not to simulate a specific period of clinical service or to establish temporal equivalence, but rather to apply an intensified and standardized hydrothermal stress capable of accelerating resin plasticization, water sorption, and silane/resin–ceramic interface degradation. Therefore, autoclave aging should be interpreted as an accelerated degradation model designed to compare the relative durability of the composite resins under severe conditions. Consequently, the SBS values observed after aging should not be considered predictors of clinical longevity, but rather indicators of material performance under intensified hydrothermal challenge, and this limitation must be acknowledged when extrapolating laboratory findings to clinical scenarios.

It should also be acknowledged that thermocycling remains the ISO-recommended method for artificial aging of dental materials in laboratory studies, as it simulates repeated thermal fluctuations between cold and hot conditions (typically 5–55°C). In this context, autoclave aging should be interpreted as a complementary accelerated degradation model rather than a direct substitute for standardized thermocycling protocols. While autoclave exposure intensifies hydrothermal stress in a shorter timeframe, it does not replicate cyclic thermal variation and therefore represents an alternative stress model rather than an equivalent simulation of clinical aging.

It is noteworthy that a high irradiance LED unit (2,700–3,000 mW/cm²) was used in the present study. High irradiance may accelerate polymerization kinetics, potentially increasing polymerization shrinkage stress at the adhesive interface. Rapid polymerization can reduce flow during the pre-gel phase, theoretically increasing interfacial stress concentration. However, the short exposure time (12 s total) was selected to balance energy delivery and minimize excessive thermal effects.

Furthermore, all specimens were polymerized under identical conditions, and no resin showed statistically inferior performance. Therefore, although increased shrinkage stress is a theoretical concern with high irradiance units, it did not negatively influence bond strength under the controlled conditions of this study.

Although Transbond XT demonstrated the highest SBS values on ceramic and Brace Paste on enamel, the absence of significant statistical differences supports the interchangeability of these materials in clinical practice. However, the substrate type and conditioning protocol remain critical factors in adhesive performance, reinforcing the importance of technique sensitivity and material compatibility in orthodontic bonding.

Although the sample size of *n* = 10 per group follows the standard adopted in many SBS studies, as documented in systematic reviews reporting that in vitro orthodontic bond strength tests commonly use 8–12 specimens per group [34, 35], the relatively high variability observed, particularly in the ceramic groups (SD ≈ 5 MPa), reduces the statistical power to detect moderate differences among composite resins. Therefore, the present study was adequately powered only to detect large effect sizes. Smaller but potentially meaningful differences may not have reached statistical significance, and the findings should be interpreted with this limitation in mind.

## Conclusions

The three composite resins evaluated demonstrated comparable SBS for orthodontic bracket bonding on enamel and resin-based CAD/CAM ceramic surfaces after accelerated aging. When appropriate surface conditioning protocols are applied, all tested materials may be considered clinically acceptable options for bonding to both substrates.

## Data Availability

The data generated and analyzed during the current study are available from the corresponding author on reasonable request.
